# Announcement: Nucleic Acids and their Interactions with Metal Ions (NAIMI)

**DOI:** 10.1155/MBD.1997.345

**Published:** 1997


					UNIVERSIT]k DEGLI STUDI DI SASSA,R,!,
NAIMI
Nucleic Acids and their
Interactions with Metal Ions
Scientific Scope
The mini symposium is intended for researchers in the Chemistry of Nucleic Acids and Metal Ions
field. The main objective of the symposium is to bring together scientists interested in the different
aspects of the influence of metal ions on the structure and metabolism of DNA. The main topics will
be DNA Polymers, nucleic metal proteins, zinc fingers and PNA.
Scientific Program
The following distinguished speakers have already agreed to deliver a lecture:
Prof. Bibudgendra Sarkar (TORONTO, CANADA),
Prof. James Allan Cowan (COLUMBUS, USA),
Prof. Bernard Meunier (CNRS, FRANCE),
Prof. Rob Kaptein (UTRECHT, THE NETHERLANDS),
Prof. Thomas W. Myers (ROCHE, USA),
Prof. Matthias W. Henteze (EMBL GERMANY),
Prof. Huguette Pelletier (HOUSTON, USA).
In addition there will be a poster session and a number of selected oral communications. All those
who wish to participate are invited to submit an abstract to the scientific committee before 31 st March
1998. The authors will be sent notification of acceptance of posters and if they have been selected
for oral presentation by 30th April 1998. The abstract must be typed in A4 format, in a single page,
centred, with 2-cm margins and with the name of the speaker underlined.
Registration
Fee before 31 March 1998
Fee after 31 March 1998
Participants: Lit. 200.000
Students: Lit. 80.000
Participants: Lit. 250.000
Students: Lit. 150.000
Registration will begin on 6 September at 3 p.m..
The Scientific Program will begin at 7 p.m. with the opening address from Prof. Sarkar.
The daily sessions will be from 9 a.m. to 7 p.m. with a break for lunch.
A social and cultural program is planned for the evenings.
To register please complete the enclosed preliminary registration form and return it by 30 June
1998.
Venue
The symposium will take place in the Centro Ricerche Porto Conte, Tramariglio, Alghero, on the
Coral Coast of North-West Sardinia with excellent beaches and the medieval city and holiday resort
Alghero about 10 Km away. The Alghero-Fertilia Airport is also 10 km from the congress centre. Bus
facilities will be available at the Airport. A map of the area and other information will be also being sent
to all participants.
Accommodation
40 single rooms are available on the campus. (price Lit. 100.000 before 31/03/98 and Lit. 150.000
after this date). Other delegates and their families will be accommodated in the nearby Porto Conte
or Capocaccia Hotels (price Lit. 170.000)
The Hotels are excellently equipped with all necessary tourist facilities such as swimming pools
recreation facilities etc.
Special program
There will be a program of social and sight seeing trips for participants and those accompanying
them. For further information please contact: NEMAPRESS Fax: +39 79 981621
For further information please contact:
Peter H. Norton Tel./Fax. +39 79 299640
e-mail ,hyperlink mailto:peter.norton @flashnet.it ,,
Valeria Maida e-mail ,,hyperlink mailto:billia@ssmain.uniss.it ,,
345

				

